# Socioeconomic differences in return to work after oncological rehabilitation: An analysis using data from German Pension Insurance

**DOI:** 10.25646/14443

**Published:** 2026-09-30

**Authors:** Enno Nowossadeck, Fabian Tetzlaff, Juliane Tetzlaff, Benjamin Barnes, Jens Hoebel

**Affiliations:** 1 Robert Koch Institute, Department of Epidemiology and Health Monitoring, Berlin, Germany; 2 Hannover Medical School, Medical Sociology Research and Teaching Unit, Hannover, Germany

**Keywords:** Return to work, Rehabilitation, Workforce, Health inequalities, Pension insurance, Germany

## Abstract

**Background:**

When employed people cannot return to work after cancer treatment, loss of income and social contacts are among the potentially precarious consequences of the disease. This article examines whether people from socioeconomically disadvantaged groups are less likely to return to work after oncological rehabilitation than people from more advantaged groups.

**Methods:**

The data source was the Scientific Use File ‘Completed rehabilitation in the insurance history’ of German Pension Insurance for 2014 – 2021. The analysis considered formal educational attainment, occupational skill level and income before rehabilitation as socioeconomic characteristics. Successful return to work was defined as being in employment for at least 50 % of weekly working hours 24 months after rehabilitation. Cox proportional hazards regression models, stratified by sex, were fitted.

**Results:**

Two years after rehabilitation, 67.2 % of people with cancer were in employment (women 70.0 %, men 63.4 %). In particular, people with lower incomes were less likely to return to work than people with higher incomes, even after controlling for other variables such as age and region of residence.

**Conclusions:**

There are socioeconomic inequalities in return to work following oncological rehabilitation, particularly to the disadvantage of people in low-income groups.

**Key messages**

► Approximately two thirds of rehabilitation patients are employed again 24 months after the end of oncological rehabilitation.
► The highest rates of return to work are found for melanoma (women and men), breast cancer (women) and cancers of the male genital organs (including prostate cancer).
► There are clear socioeconomic differences with regard to returning to work after oncological rehabilitation.
► Income is the strongest socioeconomic predictor.
► Socioeconomically disadvantaged people with cancer have a lower chance of returning to work, and the social and financial consequences affect them more severely.

## 1. Introduction

A large number of studies have shown that, in Germany as in other European welfare states and beyond, socioeconomic status is closely associated with health (e.g., [1–5]). This association generally follows a pronounced social gradient: the lower the socioeconomic position, the poorer people’s health prospects and the higher their risk of disease [1, 6]. The evidence consistently shows that this social gradient and the resulting health inequality exist across a broad spectrum of health and disease and extend to mortality and life expectancy [7–10]. Accordingly, for many common cancers such as lung, stomach and colorectal cancer, people living in socioeconomically disadvantaged circumstances also have increased risks of incidence and mortality [11–16]. However, there are exceptions among cancers: people in more socioeconomically advantaged circumstances have a higher incidence of skin cancer, while findings from Germany on breast cancer are inconsistent in this respect [11–14, 17]. Overall, the contribution of cancer to the extent and pattern of health inequality in Germany has increased over recent decades, partly because cancer trends have developed less favourably in socioeconomically disadvantaged sections of the population than in more advantaged groups [10, 12, 15]. In principle, the relationship between socioeconomic status and health arises both from an effect of socioeconomic position on health prospects and disease risks (social causation) and from causation in the opposite direction, for example through a deterioration in socio-economic status as a consequence of illness (health selection) [18]. In recent years, scientific analyses have increasingly focused on the social and financial consequences of severe chronic diseases such as cancer (e.g., [19–24]). More recent studies also focus on working age [14, 17, 25]. An analysis using data from Germany to examine trends in cancer-free working-life expectancy showed that men at older working ages and people with lower educational attainment in particular have shorter cancer-free working-life expectancy. These groups in particular showed an unfavourable relationship between increases in working-life expectancy and trends in cancer incidence and mortality over time [17]. Rehabilitation plays an important role with regard to the social and financial consequences of cancer during working age. It creates opportunities for return to work; by contrast, not commencing rehabilitation or unsuccessful rehabilitation increases the risk of reduced earning capacity, loss of income or loss of social roles.

Rehabilitation after cancer treatment is an important sector of care for cancer survivors [26]. In Germany, people with diagnosed and treated cancer have a statutory entitlement to oncological rehabilitation [27]; German Pension Insurance (DRV) is the principal benefit provider [28, 29]. The aims of oncological rehabilitation include preventing or reducing long-term impairments caused by disease and treatment [30–32]. For German Pension Insurance, return to work among employed people (RtW) is an essential task [33].

Against the background of demographic trends in Germany and increasing working-life expectancy [34–36], the return-to-work process is gaining particular relevance as a central component of tertiary prevention. The so-called baby-boomer cohorts (1959 – 1968) are currently reaching retirement age. At the same time, cancer incidence in these cohorts increases with age. The resulting reduction in the potential labour force [37] could be mitigated if more people with cancer could be kept in employment after completion of medical care [38]. In addition, RtW has a positive effect on the physical and mental health of oncology patients [39].

Two strands can be distinguished in the literature on RtW after cancer. The first, which accounts for the majority of these publications, analyses RtW after cancer regardless of whether patients received oncological rehabilitation. Studies examine RtW after breast cancer particularly frequently, which is reasonable given the comparatively low mean age at diagnosis (65 years, [40]). Comparing RtW rates across cancer types is difficult because 1-year survival rates can differ substantially by cancer type. The 1-year survival rate is 96 % for breast cancer, whereas for lung cancer it is 53 % in women and 46 % in men [41]. We should therefore expect marked differences in RtW rates between cancer types.

Studies analyse RtW after oncological rehabilitation far less frequently for cancer overall or for other individual cancer types. In Germany, a prospective clinical study in four rehabilitation facilities found that around three quarters of cancer survivors who had undergone oncological rehabilitation had returned to work 12 months after rehabilitation [42].

This article examines whether people from socioeconomically disadvantaged groups are less likely to return to work after oncological rehabilitation than people from more socioeconomically advantaged groups. The analyses are conducted for cancer overall and for important individual cancer types.

## 2. Methods

### 2.1 Data

The analyses presented in this paper are based on data from the Scientific Use File ‘Completed rehabilitation in the insurance history’ of the German Pension Insurance Research Data Centre (FDZ-RV SUF) for 2014 – 2021 [43]. It contains a 20 % sample of all people insured by German Pension Insurance who completed at least one medical rehabilitation service during the reporting period (N = 1,178,600). We identified cancer on the basis of a confirmed principal diagnosis according to ICD-10 (C00 – C97).

In Germany, following acute oncological treatment (such as surgery, chemotherapy or radiotherapy), there is an entitlement to oncological rehabilitation at an appropriate rehabilitation facility. Such rehabilitation can only be provided if there is a prospect of success. For German Pension Insurance, return to work is an important rehabilitation goal [33]. However, analyses must take into account a particular feature of oncological rehabilitation compared with rehabilitation for other disease entities. German Pension Insurance normally provides rehabilitation for contributors to the statutory pension insurance scheme, but not for benefit recipients (for example, recipients of an old-age pension), for whom statutory health insurance is responsible. There is a statutory exception for oncological rehabilitation. Pursuant to Section 31(1) no. 2 of Book VI of the German Social Code (SGB VI), family members of contributing DRV-insured persons and pension recipients may also take part in an oncological rehabilitation programme irrespective of their insurance status or age.

Stratified by biological sex and cancer type, the analysis examines whether people with cancer from socioeconomically disadvantaged groups have lower rates of RtW than people from more advantaged groups. Successful return to work is defined as being in employment for at least 50 % of usual weekly working hours within 24 months after the end of the rehabilitation programme.

The socioeconomic characteristics considered were formal education, occupational position and income of the insured persons:

► formal educational attainment (based on the consolidated education variable of German Pension Insurance [43], available in the categories low, medium and high)
► occupational skill level (grouped as unskilled and semi-skilled occupations, skilled occupations, complex specialist occupations and highly complex occupations)
► income before rehabilitation (grouped as low (1st quintile), medium (2nd to 4th quintile) and high (5th quintile)).

### 2.2 Statistical methods

To analyse socioeconomic inequalities in RtW after oncological rehabilitation, we calculated age-standardised RtW rates by socioeconomic group. The population of Germany according to the 2011 Census served as the standard population for direct age standardisation. In the multivariable analysis, we used event history analysis methods to control for other variables. We applied Cox proportional hazards regression models stratified by sex and cancer type. We controlled for age at the start of rehabilitation (18 – 65 years; beginning with the under-25 age group and subsequently in 5-year age groups). We entered the three socioeconomic indicators into the models simultaneously, i.e. we mutually adjusted education, occupation and income. Finally, we included additional contextual variables, such as rehabilitation year (year in which rehabilitation ended) and region of residence (Eastern Germany incl. Berlin/Western Germany), as independent variables to complete the full regression model. We excluded people who died during or after rehabilitation or started receiving a pension (full and partial reduction in earning capacity of more than 50 %) from the analyses from that time point onwards (right censoring). Estimates are reported with 95 % confidence intervals.

## 3. Results

### Characteristics of the study population

The Scientific Use File from the German Pension Insurance contained 221,461 oncological rehabilitation cases for 2014 – 2020. Of these, 67.6 % were excluded because they were not in employment or were employed for less than 50 % of weekly working hours (see Figure 1). This left 71,756 people aged 18 – 65 years who had been in employment for at least 50 % of weekly working hours before rehabilitation. A further 7,676 people were excluded because no confirmed cancer diagnosis (according to the 10th Revision of the International Statistical Classification of Diseases and Related Health Problems, ICD-10) was documented. Thus, data from 64,080 people with a confirmed cancer diagnosis were available for analysis (36,018 women, 28,062 men; see Table 1).

Around three quarters of oncological rehabilitation patients were aged 50 years or older (76.6 %; women 72.6 %; men 81.8 %). Table 1 provides further information on formal educational attainment, occupational skill level, income and region of residence.

#### Return to Work

Around two thirds of oncological rehabilitation patients who were in employment before rehabilitation were again in employment for at least half of normal working hours two years after rehabilitation (43,015 people; non-age-standardised 67.2 %). Among women, this was 25,221 people (non-age-standardised 70.0 %) and among men 17,794 (non-age-standardised 63.4 %).

Table 2 shows the age-standardised rates of successful return to work among all rehabilitation patients, differentiated by cancer diagnosis. Malignant melanoma overall (both sexes), breast cancer (women) and cancers of the male genital organs (including prostate cancer) have the highest RtW rates. Women have the lowest RtW rates for cancers of the oesophagus/stomach and liver/biliary tract, while men have the lowest rates for cancers of the oesophagus/stomach and respiratory organs (including lung).

Table 3 provides initial indications of socioeconomic differences in RtW rates. The age-standardised rate of women with low formal educational attainment who had returned to work was 60.5 %, compared with 77.3 % among women with high formal educational attainment. Among men, age-standardised RtW rates are somewhat lower in all education groups but show the same pattern. As occupational skill level increases, age-standardised RtW rates rise among both women and men; among women, for example, from 57.3 % (unskilled and semi-skilled occupations) to 78.5 % (highly complex occupations). Income shows a similar pattern: in the high-income group, 89.4 % of women and 85.6 % of men had returned to work two years after rehabilitation, compared with only 58.4 % of women and 41.9 % of men in the low-income group.

These descriptive analyses therefore show that, for cancer overall, RtW rates in the higher subgroups of all three socioeconomic indicators (higher educational attainment, higher occupational skill level, higher income) are higher than in the overall sample among both women and men.

The Cox regression results provide clear findings for cancer overall (see Figure 2). After adjustment for age, rehabilitation year, region of residence and the other socioeconomic indicators, women and men with low or medium educational attainment have lower RtW rates (hazard ratio below 1) than women and men with high educational attainment. Women and men in unskilled and semi-skilled occupations also have a lower rate of return to work than people in the reference category of highly complex occupations. Income likewise shows a social gradient: women and men with low or medium income before rehabilitation have a markedly lower rate of successful RtW than women and men with high income. In low-income groups, RtW rates (at the same level of education and occupational position) are only about half as high as in the high-income group, with the difference more pronounced among men than women.

The analysis results for the cancer types examined (see Figure 3) show a somewhat less clear pattern with regard to inequalities by education and occupational groups. The confidence intervals of the hazard ratios are often very wide, so that differences between the groups of the socioeconomic indicators ‘formal educational attainment’ and ‘occupational skill level’ are apparent but not statistically significant. Exceptions among women are breast cancer and kidney/bladder cancer (educational attainment). Among men, there are significant differences between the lower or medium group and the reference group for colorectal cancer (educational attainment and occupational skill level), mouth/pharyngeal cancer (occupational skill level), oesophageal/stomach cancer (educational attainment), male genital organ cancer, kidney/bladder cancer and liver cancer (occupational skill level).

The social gradient is much clearer when operationalised using income before rehabilitation. For all cancer types analysed, the rate of successful return to work two years after rehabilitation is significantly lower in the low-income group than in the group with high average earnings before rehabilitation, both among women (except liver cancer) and among men.

### 4. Discussion

Within German Pension Insurance, between 2014 and 2020, RtW rates two years after oncological rehabilitation were 70.0 % for women and 63.4 % for men. For all three socioeconomic indicators, RtW rates were higher in the highest socioeconomic groups than in the lowest groups, among both women and men.

Studies using examination and survey data also found similarly high RtW rates. Mehnert and Koch reported a RtW rate of 75.7 % among 750 people with cancer in a prospective study [42]. In a randomised intervention study examining the effectiveness of work-related medical rehabilitation for cancer among 484 cancer survivors, around 75 % of both the intervention and control groups had returned to work one year after oncological rehabilitation [44]. In another study, Fauser et al. reported ‘5 to 6 out of 10 affected people in stable occupational participation’ two years after oncological rehabilitation in 2017 according to the German Pension Insurance [45, p. 108].

Age-standardised RtW rates differ markedly by cancer type, ranging from around 45 % to just under 80 %. The available data do not allow conclusions about the factors influencing these differences.

In their 2023 study, Fauser et al. report rates of stable employment two years after rehabilitation for four specific cancers (breast, colorectal, lung, prostate) [45]. These rates are close to the RtW rates calculated here. These rates are lower than the age-standardised RtW rates in Table 2, but the relative pattern across cancer types is similar: breast cancer and cancers of the male genital organs (including prostate cancer) have the highest rates, followed by colorectal cancer and cancers of the respiratory organs (including lung cancer).

When comparing these results with those of other studies, it should be noted that such comparisons are of limited interpretability because return to work is often operationalised differently.

The descriptive analyses showed that, for each of the three socioeconomic indicators, RtW rates differ across socioeconomic groups such that the highest socioeconomic group has higher RtW rates than the lower groups.

After mutual adjustment for the socioeconomic indicators, income is the strongest socioeconomic predictor of differences in RtW rates, as Figure 3 clearly illustrates. With the exception of liver cancer among women, hazard ratios for the low-income group are substantially lower than for the high-income group across all cancer types.

The rehabilitation dataset used here does not permit empirical explanations for the observed differences. Socioeconomically determined differences in cancer do not arise only in rehabilitative care; rather, they accumulate across the entire preceding course of disease and the care pathway (‘cancer care continuum’). For example, socioeconomic differences in risk behaviour (e.g., tobacco use), early detection (participation in screening programmes) or diagnosis (tumour size and stage) can affect the entire subsequent course [46, 47]. Participation outcomes ultimately reflect these cumulative effects, which oncological rehabilitation evidently cannot sufficiently offset.

RtW studies in the German rehabilitation system frequently include socioeconomic variables in their analyses (e.g., [42, 45]); however, studies in Germany that systematically analyse socioeconomic differences while considering all socioeconomic indicators (measured here by formal educational attainment, occupational skill level and income) remain scarce. One of the few studies from Germany to take this approach likewise found that people with low income, lower educational attainment or lower occupational position have a lower probability of returning to work after medical rehabilitation [48]. Additional analyses in that study also found this for people in the diagnostic group ‘neoplasms’, although the authors did not further break this group down by cancer diagnosis.

RtW is an important means of coping with the health and social consequences of cancer among employed people [49]. Successful return to work also has several positive effects. At the individual level, returning to work prevents substantial loss of income and the loss of social connectedness and identity-conferring roles in working life [22]. ‘Financial toxicity’ warrants particular attention [21, 50]: if people with cancer who have a low income before rehabilitation have a lower chance of returning to work for health reasons, the financial losses are all the more serious and may constitute a substantially increased risk of poverty. At the institutional level, people with cancer who return to work remain contributors to German Pension Insurance; otherwise, they become benefit recipients (reduced earning capacity pension) [33]. At the societal level, successful RtW thus avoids transfer payments and a disease-related reduction in the potential labour force, which is highly important given demographic ageing [37].

#### Strengths and limitations

The strengths of this study derive from the data used from the German Pension Insurance. The sample size and the use of data on RtW after rehabilitation are particularly important. As a sample from German Pension Insurance claims data, the dataset enables detailed analyses not only for cancer overall but also for different cancer types because of its size. Second, there is no participation bias as in clinical studies. Third, unlike the rehabilitation statistics of the Federal Statistical Office, the dataset contains information on all three core dimensions of socioeconomic position (education, occupation, income).

Limitations include the lack of information on the individual attitudes of rehabilitation patients, which could provide insight into health-related and non-health-related factors and reasons for not returning to work.

#### Conclusions

There is substantial socioeconomic inequality in return to work after oncological rehabilitation, particularly to the disadvantage of people with cancer in low-income groups. The socioeconomic differences in the course and consequences of cancer that arise throughout the disease and treatment pathway evidently cannot be offset by the existing rehabilitation system. The results therefore point to a double disadvantage for people with cancer from socioeconomically disadvantaged groups after rehabilitation: their chances of successful return to work are lower than those of people with cancer from more socioeconomically advantaged groups. In addition, the social and financial consequences of not being in employment after rehabilitation, such as reduced earning capacity, loss of income or loss of social roles, weigh particularly heavily on people with cancer from socioeconomically disadvantaged groups and constitute a substantially increased risk of poverty for those affected. One instrument developed and established within German Pension Insurance to improve return to work is work-related medical rehabilitation (MBOR, [51]). It addresses occupational problems resulting from health limitations. It is clear that both MBOR and ‘transition management into active employment’ [52] should be geared towards the needs of people with cancer from socioeconomically disadvantaged groups in particular.

## Figures and Tables

**Figure 1:**
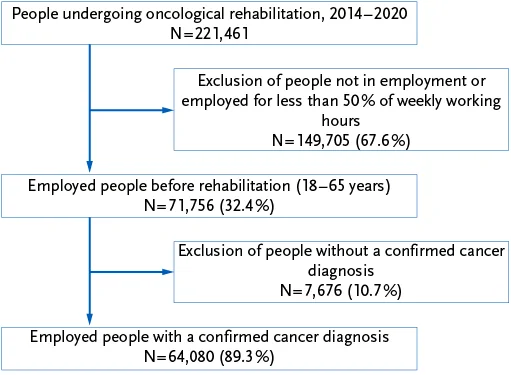
Flow chart for inclusion and exclusion of rehabilitation patients. Source: FDZ-RV SUF

**Figure 2:**
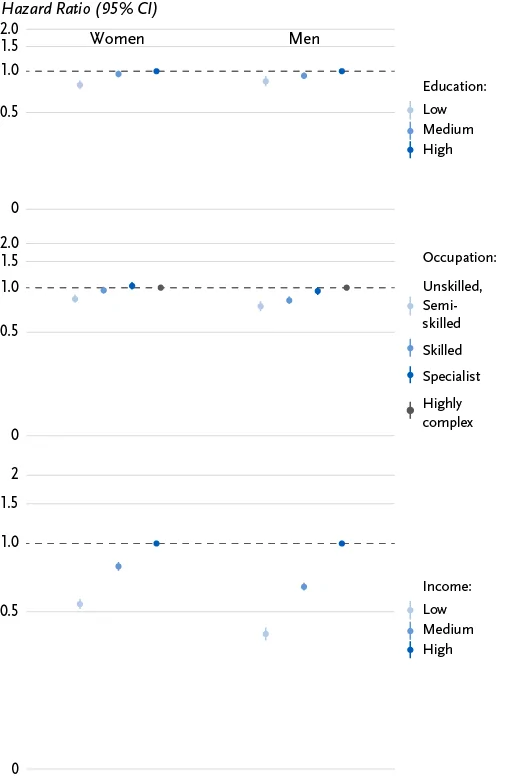
Results of Cox regressions for return to work by socioeconomic position for cancer overall (adjusted hazard ratios), by sex, 2014 – 2021 (n = 36,018 women, n = 28,062 men). Source: FDZ-RV SUF Own calculations Reference categories: the group with the highest level in each case Note: Hazard ratios are adjusted for age, region of residence, rehabilitation year and the respective other socioeconomic indicators (mutual adjustment for education, occupation and income) CI = confidence interval

**Figure 3:**
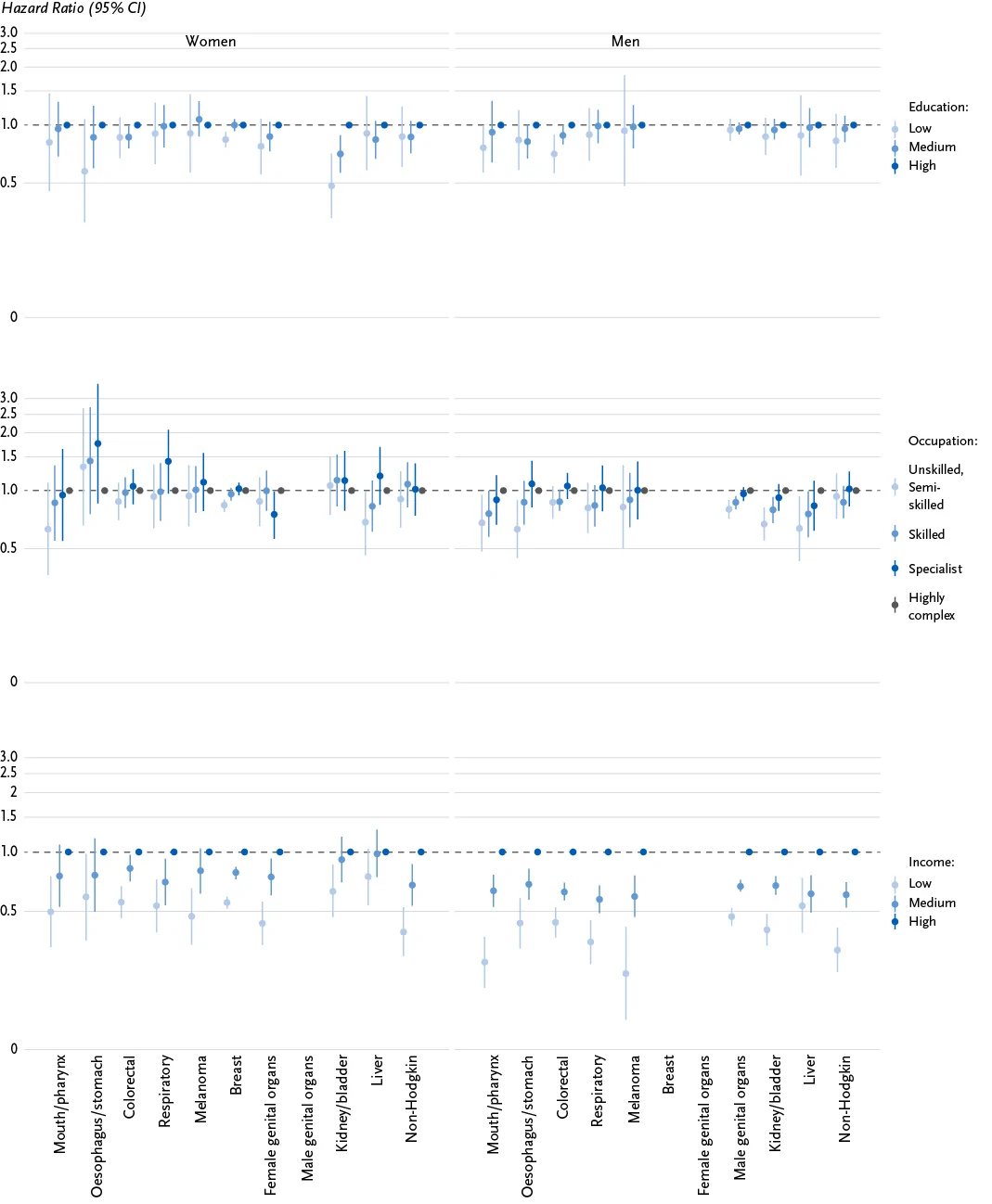
Results of Cox regressions for return to work by socioeconomic position for different cancer types (adjusted hazard ratios), by sex, 2014 – 2021 (n = 36,018 women, n = 28,062 men). Source: FDZ-RV SUF Own calculations Reference categories: the group with the highest level in each case Note: Hazard ratios are adjusted for age, region of residence, rehabilitation year and the respective other socioeconomic indicators (mutual adjustment for education, occupation and income) CI = confidence interval

**Table 1:** Characteristics of the study population by sex, 2014 – 2020 (n = 36,018 women, n = 28,062 men). Source: FDZ-RV SUF

	**Women**		**Men**		**Overall**	
	**n**	**%**	**n**	**%**	**n**	**%**
	**36,018**	**100**	**28,062**	**100**	**64,080**	**100**
**Age**						
< 25	117	0.3	169	0.6	286	0.4
25 – 29	344	1.0	342	1.2	686	1.1
30 – 34	770	2.1	543	1.9	1,313	2.0
35 – 39	1,309	3.6	694	2.5	2,003	3.1
40 – 44	2,325	6.5	1,068	3.8	3,393	5.3
45 – 49	4,995	13.9	2,282	8.1	7,277	11.4
50 – 54	9,338	25.9	5,227	18.6	14,565	22.7
55 – 59	9,452	26.2	8,682	30.9	18,134	28.3
60 – 65	7,368	20.5	9,055	32.3	16,423	25.6
**Rehabilitation year**						
2014	3,923	10.9	3,242	11.6	7,165	11.2
2015	4,458	12.4	3,665	13.1	8,123	12.7
2016	4,668	13.0	3,612	12.9	8,280	12.9
2017	4,833	13.4	3,894	13.9	8,727	13.6
2018	5,586	15.5	4,257	15.2	9,843	15.4
2019	6,378	17.7	4,763	17.0	11,141	17.4
2020	6,173	17.1	4,612	16.4	10,785	16.8
**Formal education**						
Low	1,942	5.4	1,514	5.4	3,456	5.4
Medium	23,237	64.5	16,844	60.0	40,081	62.5
High	7,522	20.9	6,433	22.9	13,955	21.8
Qualification unknown	3,317	9.2	3,271	11.7	6,588	10.3
**Occupational skill level**						
Unskilled and semi-skilled occupations	5,919	16.4	3,314	11.8	9,233	14.4
Skilled occupations	21,736	60.3	15,940	56.8	37,676	58.8
Complex specialist occupations	4,023	11.2	4,076	14.5	8,099	12.6
Highly complex occupations	3,650	10.1	3,440	12.3	7,090	11.1
Not classifiable	690	1.9	1,292	4.6	1,982	3.1
**Average earnings before rehabilitation**						
Low	9,990	27.7	3,657	13.0	13,647	21.3
Medium	21,988	61.0	16,045	57.2	38,033	59.4
High	4,040	11.2	8,360	29.8	12,400	19.4
**Region of residence**						
Eastern Germany	9,355	26.0	6,776	24.1	16,131	25.2
Western Germany	26,663	74.0	21,286	75.9	47,949	74.8

Own calculations

**Table 2:** Age-standardised rates of return to work (%) by sex, 2014 – 2020 (n = 36,018 women, n = 28,062 men). Source: FDZ-RV SUF

	**Women** **%**	**Men** **%**
All cancers	71.2	65.9
Mouth and pharynx – C00 – 14	62.1	66.2
Oesophagus, stomach – C15 – 16	45.9	46.9
Colon and rectum – C17 – 21	68.2	64.9
Respiratory organs – C30 – 39	60.1	43.3
Melanoma – C43 – 44	79.9	76.6
Breast – C50	77.3	–
Vulva, Vagina, Cervix uteri – C51 – 53	68.6	–
Male genital organs – C60 – 63	–	79.5
Urinary organs – C64 – 68	72.4	76.6
Liver/biliary tract – C22 – 26, C76 – 80, C97	46.7	53.4
Non-Hodgkin diseases – C82 – 90	66.1	64.4

Own calculations; standard population: Germany 2011

**Table 3:** Return to Work: Numbers and age-standardised rates by sex and formal educational attainment, occupational skill level and income before rehabilitation, 2014 – 2020 (n = 36,018 women, n = 28,062 men). Source: FDZ-RV SUF

	**Women**		**Men**	
	**n**	**% (95 % CI)**	**n**	**% (95 % CI)**
Overall	25,221	100	17,794	100
**Formal education**				
Low	1,158	60.5 (50.8 – 70.7)	839	55.4 (48.2 – 63.0)
Medium	16,364	71.2 (68.3 – 74.2)	10,553	67.2 (64.4 – 70.1)
High	5,819	77.3 (72.3 – 82.3)	4,783	71.9 (67.7 – 76.3)
Missing	1,880		1,619	
**Occupational skill level**				
Unskilled and semi-skilled occupations	3,522	57.3 (51.6 – 63.1)	1,737	55.8 (50.4 – 61.3)
Skilled occupations	15,431	72.1 (69.2 – 75.0)	9,715	63.3 (60.8 – 66.0)
Complex specialist occupations	3,092	78.5 (70.5 – 86.7)	2,994	75.0 (67.8 – 82.2)
Highly complex occupations	2,803	78.5 (67.8 – 89.4)	2,676	85.4 (73.5 – 97.6)
Missing	373		672	
**Income**				
Low	5,689	58.4 (55.5 – 61.4)	1,499	41.9 (38.7 – 45.3)
Medium	16,170	79.5 (74.5 – 84.7)	9,608	69.1 (65.4 – 72.8)
High	3,362	89.4 (61.9 – 117.6)	6,687	85.6 (77.4 – 94.0)

Own calculations, standard population: Germany 2011

CI = confidence interval
